# 1962. Perceptions of the COVID-19 Vaccine within the Sudanese-American Community

**DOI:** 10.1093/ofid/ofac492.1587

**Published:** 2022-12-15

**Authors:** Jonathan R Freese, Harlan R Sayles, Fatima Ahmed, Mujtaba Abdellatif, Nada Fadul

**Affiliations:** University of Nebraska-Medical Center, Omaha, Nebraska; University of Nebraska Medical Center, Omaha, Nebraska; n/a, New York City, New York; n/a, New York City, New York; University of Nebraska Medical Center, Omaha, Nebraska

## Abstract

**Background:**

One of the primary issues affecting COVID-19 vaccine uptake in high-income countries is vaccine hesitancy, which is prevalent in people from different countries of origin. Characterizing vaccine uptake in immigrant and refugee populations in the US could provide a unique window into both local and global health behaviors. The goal of this project is to characterize Sudanese American perspectives on the COVID-19 vaccine.

**Methods:**

We conducted an anonymous, online, anonymous, cross-sectional survey directed toward Sudanese Americans, with survey development guided by principles from the Vaccine Examination Scale. The survey was distributed in both English and Arabic and included questions about vaccination history, motives for getting the vaccination, reasons for vaccine hesitancy, and barriers to vaccination. Fisher’s exact tests were used to analyze evaluate possible associations between vaccine uptake and sources of information on the vaccine and social media use, respectively. Data analysis was conducted using STATA SE v17.0 (StataCorp, College Station, TX).

**Results:**

A total of 108 survey responses were received; 4 were excluded for failing to meet inclusion criteria. A total of 92% received at least one dose of COVID-19 vaccine, with the primary motivation being to protect oneself (62%). Only 8 had not been vaccinated and, of those, 2 were willing to take the vaccine. Of the 6 unwilling to take the vaccine, the most cited reason was a belief that it had not been studied enough. Of the 14 possible hesitancy responses, 9 were selected at least once.

When asked about their primary source of information on COVID-19, 44% used government websites, followed by mass media (22%), social media (12%) and health personnel (11%). Using Fisher’s exact tests, no statistically significant conclusions were drawn between vaccine uptake and primary source of information (P = .097) or specific types of social media.

**Conclusion:**

Vaccine uptake among our survey population (92%) was much higher than that of the US (77%) or Sudanese population (11%). Overall motivators for vaccine hesitancy and vaccine uptake varied and no specific correlations were found to be associated to vaccine uptake. Future research should evaluate high levels of vaccine uptake in this community.

**Disclosures:**

**All Authors**: No reported disclosures.

